# Editorial Comment: Intra-cavernous injection of BOTOX ® (50 and 100 Units) for treatment of vasculogenic erectile dysfunction: Randomized controlled trial

**DOI:** 10.1590/S1677-5538.IBJU.2022.06.03

**Published:** 2022-11-01

**Authors:** Rodrigo de Castro Barros

**Affiliations:** 1 Universidade Federal Fluminense Hospital Universitário Antônio Pedro Niterói RJ Brasil Serviço de Urologia, Hospital Universitário Antônio Pedro - Universidade Federal Fluminense - UFF, Niterói, RJ, Brasil

Waleed El-Shaer ^1^, Hussein Ghanem ^2^, Tamer Diab ^1^, Ahmed Abo-Taleb ^1^, Wael Kandeel ^1^

**Andrology. 2021 Jul;9(4):1166-1175.**

**DOI: 10.1111/andr.13010 | ACCESS: 33784020**

## COMMENT

Onabotulinum toxin-A (BTX) is widely used to treat various medical conditions such as striated and smooth muscle disorders. In urologic disorders, the use of BTX has been approved to treat detrusor overactivity (1).

Despite the various therapeutic options for the treatment of erectile dysfunction (ED), there is a need for a new effective and safe treatment for patients who are refractory to noninvasive therapies. Some studies have suggested the possible role of intracavernosal injection (ICI) of BTX for the treatment of ED, which has aroused interest in the area of sexual medicine (2–4).

BTX inhibits sympathetic adrenergic or cholinergic vasoconstriction, sensory nerves, decreasing the tone of penile resistance vessels, blood flow, and cavernosal smooth muscle tone, which are involved in the pathophysiology of DE (5).

In this interesting study, the authors compared the safety, efficacy and durability of ICI of BTX at different doses (50 and 100μ) against placebo (saline) in the management of vasculogenic ED non-responsive to pharmacological therapy (phosphodiesterase type 5 inhibitors and/or ICI of trimix). They conducted a prospective randomized double-blind placebo-controlled trial involving 176 patients, who were randomly assigned to one of the treatments. All patients were monitored for six months. Significant improvements in all parameters (Sexual Health Inventory for Men Score, Erection Hardness Score, Sexual Encounter Profile, Global Assessment Score, and Doppler parameters) were observed in patients in the BTX-100μ and BTX-50u groups, with maximum improvement in the third month of treatment. BTX-100U was more durable with statistically significant difference between the aforementioned groups in favor of BTX-100U. They observed some adverse events, such as injection site penile pain and hematoma. Interestingly, four patients developed prolonged sustained erection during penile Doppler examination at three months, which was managed conservatively, and at six months, one patient from the BTX-100μ group had priapism, which was resolved by ICI of ephedrine. On the other hand, no systemic side effects were observed. According to the authors, BTX can promote a safe, effective and relatively durable improvement of ED.

The present study has some limitations, such as failure to stratify the different causes of vasculogenic ED and measure psychological problems that can have an important impact on the response to treatment. In addition, the study was conducted in a single institution and with a short follow-up. However, the authors presented one of the pioneering works evaluating the effects ICI of BTX on the treatment of ED. Congratulations to the authors for their contribution with this important and interesting report.

ICI of BTX is a promising second-line treatment option of ED. However, unfortunately we have more questions than answers on this topic. Which profile makes patients candidates for this therapy? Is long-term ICI of BTX safe? What is the ideal dose to start? What is the duration of the effect of BTX on erectile function? Thus, larger-scale human multicenter prospective randomized double-blind placebo-controlled studies with a long follow-up period are needed to determine the therapeutic efficacy and clinical safety of BTX for the treatment of ED.
